# Risk Stratification and Factors Associated with Heart Failure Hospitalization During Osimertinib Therapy

**DOI:** 10.3390/curroncol33100586

**Published:** 2026-09-29

**Authors:** Tomoya Sugimoto, Yoshihiro Noguchi, Rikuto Masuda, Sorami Tani, Tomohiko Harada, Yukio Toyama, Mitsuru Saguchi, Tomoaki Yoshimura

**Affiliations:** 1 Endowed Course of Advanced Medical Care Community Pharmacy, Gifu Pharmaceutical University, 1-25-4 Daigakunishi, Gifu-shi 501-1196, Gifu, Japan; tomoya_sugimoto_aa@mail.toyota.co.jp (T.S.); saguchi@chubuyakuhin.co.jp (M.S.); 2 Department of Pharmacy, Toyota Memorial Hospital, 1-1 Heiwa-cho, Toyota-shi 471-8513, Aichi, Japan; yukio_toyama@mail.toyota.co.jp; 3 Laboratory of Clinical Pharmacy, Gifu Pharmaceutical University, 1-25-4 Daigakunishi, Gifu-shi 501-1196, Gifu, Japan; noguchiy@gifu-pu.ac.jp (Y.N.); 205101@gifu-pu.ac.jp (R.M.); 225065@gifu-pu.ac.jp (S.T.); harada.1d70c@kanagawa-pho.jp (T.H.); 4 Department of Pharmacy, Kanagawa Prefectural Ashigarakami Hospital, 866-1 Matsudasoryo, Matsuda-machi, Ashigarakami-gun 258-0003, Kanagawa, Japan

**Keywords:** osimertinib, heart failure hospitalization, EGFR-TKI, nomogram, risk stratification, cardio-oncology, competing risks

## Abstract

Background: Osimertinib is the standard of care for epidermal growth factor receptor mutation-positive non-small cell lung cancer, but heart failure hospitalization (HFH) remains a concern, and no practical tool exists to stratify HFH risk during therapy. Methods: In this retrospective cohort study using a Japanese claims database, 5802 patients newly initiating osimertinib were followed for HFH during treatment. Baseline predictors assessed before the index date were selected by 10-fold cross-validated least absolute shrinkage and selection operator (LASSO) logistic regression and internally validated by bootstrapping (B = 500), repeating predictor selection in each resample. Cumulative incidence functions and Fine–Gray models treated death as a competing event; cause-specific Cox models served as sensitivity analyses. Results: HFH occurred in 169 patients (2.9%) during a median on-treatment follow-up of 9.3 months, with 268 competing deaths. LASSO retained seven predictors. The apparent concordance index (C-index) was 0.731 (95% confidence interval, 0.691–0.771) and the optimism-corrected C-index 0.718. Observed incidence increased 10.2-fold across predicted-risk deciles (1.03–10.50%). Fine–Gray and Cox estimates closely matched the logistic model. Conclusions: Baseline cardiovascular vulnerability stratifies HFH risk during osimertinib therapy. The nomogram, not externally validated, is best used as a screening aid to identify patients who may benefit from intensified cardiovascular monitoring.

## Simple Summary

Osimertinib is a widely used and highly effective oral therapy for lung cancer driven by specific genetic alterations. Although it is generally well tolerated, some patients are hospitalized for heart failure during treatment, and doctors currently have no simple means of identifying those at risk in advance. Using a large real-world health database, we found that older age, previous hospitalization for heart failure, an irregular heartbeat, and the use of certain heart medications before treatment were associated with these hospitalizations. We combined these factors into a simple scoring chart. This chart cannot replace cardiac ultrasound scans or blood tests, and our data cannot establish that osimertinib itself caused the hospitalizations. Because the chart separated patients into groups with an approximately tenfold difference in risk, it is most useful as a screening aid for identifying patients who require closer assessment of heart function rather than for predicting an individual patient’s risk.

## 1. Introduction

Osimertinib, a third-generation epidermal growth factor receptor (EGFR) tyrosine kinase inhibitor (TKI), plays a pivotal role in the treatment of EGFR mutation-positive non-small cell lung cancer (NSCLC). It was developed to target the T790M mutation, a common mechanism of resistance to first- and second-generation EGFR-TKIs, and was introduced as a therapeutic option following the development of resistance [1]. Subsequently, the FLAURA trial demonstrated its efficacy as first-line therapy by showing prolonged progression-free and overall survival [2,3]; osimertinib is now widely used as the standard-of-care treatment for advanced EGFR mutation-positive NSCLC. Moreover, the ADAURA trial established the efficacy of osimertinib in the adjuvant setting [4,5], extending its indications from early-stage to advanced lung cancer.

Osimertinib exhibits excellent central nervous system penetration and provides substantial therapeutic benefits to patients with brain metastases [6]. It is generally well tolerated and is associated with a lower incidence of adverse events, such as skin toxicity and diarrhea, than conventional EGFR-TKIs [2,6]. Owing to its pronounced efficacy and favorable tolerability, osimertinib is widely used as the standard treatment for EGFR mutation-positive NSCLC [3,4,5].

However, concerns regarding osimertinib-associated cardiovascular adverse events have increased with its expanding use. Although off-target effects mediated through the human epidermal growth factor receptor 2 (HER2) signaling pathway have long been postulated, more recent studies have identified mechanisms of cardiotoxicity not previously observed with other anticancer agents, including mitochondrial dysfunction and sarcomeric impairment mediated through the GATA4-MYLK3-MYL2 axis [7,8,9,10]. Cardiovascular adverse events, including heart failure, cardiomyopathy, left ventricular dysfunction, arrhythmia, QT prolongation, and myocarditis, have been documented in clinical trials, post-marketing surveillance, spontaneous-reporting database analyses, and real-world data analyses [11,12].

Among these adverse events, heart failure hospitalization (HFH) is a particularly important clinical outcome because it not only compromises prognosis and quality of life but also contributes to treatment interruption and increased healthcare resource utilization [13]. Patients receiving osimertinib are often older adults with pre-existing cardiovascular comorbidities, such as hypertension, ischemic heart disease, and atrial fibrillation; therefore, the early identification of patients at high risk of HFH is crucial for facilitating appropriate monitoring and intervention. Although the incidence and clinical features of osimertinib-associated cardiovascular adverse events have been reported [14], the independent predictors of HFH during osimertinib therapy and a practical, integrated risk assessment tool have not been sufficiently established.

In the present study, we used a large-scale real-world database to identify baseline factors associated with HFH during osimertinib therapy, construct a nomogram for risk stratification, and evaluate the nomogram within a time-to-event framework accounting for death as a competing event. Predictors were selected using penalized least absolute shrinkage and selection operator (LASSO) regression [15,16]. Because all patients received osimertinib and no comparator cohort was available, this analysis represents risk stratification within an osimertinib-treated population rather than an assessment of cardiotoxicity attributable to osimertinib.

## 2. Materials and Methods

### 2.1. Study Design and Data Source

This retrospective observational study used the DeSC database (DeSC Healthcare, Inc., Tokyo, Japan), a large-scale administrative claims and health check-up database established in Japan in 2020. Data from April 2014 to March 2023 were used. Patients were eligible if they had newly initiated osimertinib therapy for lung cancer (International Classification of Diseases, 10th Revision [ICD-10] code C34), and the date of the first prescription was defined as the index date. Index dates from May 2016 to March 2023 were eligible, and the end of March 2023, the last month of the available data, was the administrative end of follow-up. A look-back period of at least six months before the index date was required to characterize baseline comorbidities and concomitant medications. Patients younger than 18 years at the index date were excluded. The study was designed, analyzed, and reported in accordance with the Transparent Reporting of a multivariable prediction model for Individual Prognosis Or Diagnosis (TRIPOD) statement for the development and validation of multivariable prediction models [17]; the completed checklist is provided as Table S1.

Patients were followed from the index date until the earliest occurrence of any of the following events: (1) HFH; (2) a change in drug class (from osimertinib to another EGFR-TKI or chemotherapy); (3) the last prescription date plus 30 days; or (4) death. The primary endpoint was HFH during follow-up. Drug exposure-related events were defined as those occurring from seven days after the index date to 30 days after the last prescription date. These criteria restricted outcome ascertainment to the on-treatment period. The date of death was derived from the month in which insurance eligibility was lost for patients flagged as deceased; because the database records dates by calendar month, the last day of that month was used. Patients flagged as deceased but with an uninterpretable eligibility end date were censored at the end of the observable period. Loss of insurance eligibility for reasons other than death (e.g., relocation, transfer to another health insurance system, or initiation of public assistance) was treated as censoring at the time eligibility was lost, because the same individual cannot be followed under a different insurer.

### 2.2. Primary Endpoint and Definition of Variables

The primary endpoint, HFH, was defined as (i) a heart failure diagnosis code recorded as the reason for admission or the principal diagnosis (ICD-10 I50.0, I50.1, I50.9, or I11.0; suspected diagnoses were excluded), together with (ii) the administration of intravenous furosemide, a vasodilator (nitroglycerin, isosorbide dinitrate, carperitide, or nicorandil), or an inotrope (dobutamine, dopamine, noradrenaline, milrinone, or olprinone) within 2 days of the heart failure diagnosis. The requirement for intravenous treatment was based on the acute heart failure algorithm validated against chart reviews of Japanese claims data by Kanaoka et al. [18] (ICD-10 I50 or I11.0 with an intravenous diuretic administered within 2 days of admission; sensitivity, 0.74; positive predictive value, 0.79). We additionally required the heart failure code to be included among the admission diagnoses to ensure that heart failure arising during an admission for another reason was not counted as an event. Prior HFH was defined as at least one HFH, identified using the same algorithm, at any time before the index date and was coded as present or absent.

Comorbidities at the index date included prior HFH, hypertension, diabetes mellitus, dyslipidemia, ischemic heart disease, atrial fibrillation/other arrhythmias, cardiomyopathy, valvular disease, chronic kidney disease, chronic obstructive pulmonary disease, alcohol-related mental and behavioral disorders, and alcoholic liver disease, as identified from recorded diagnoses. Diagnosis records with a “suspected” flag, which is entered in Japanese claims to justify diagnostic testing rather than document an established disease, were excluded, and at least one qualifying record during the look-back period was required. Atrial fibrillation and other arrhythmias were analyzed as a single category because the rhythm-specific codes were too sparse to be modeled separately. All predictors were ascertained during the look-back period and fixed at the index date.

Baseline heart failure-related medications included beta-blockers, angiotensin-converting enzyme (ACE) inhibitors, angiotensin II receptor blockers (ARBs), mineralocorticoid receptor antagonists (MRAs), sodium–glucose cotransporter 2 (SGLT2) inhibitors, loop diuretics, and tolvaptan. Medication exposure was defined as at least one prescription during the 180 days before the index date, and oral loop diuretic use was distinguished from intravenous use. In addition, concomitant platinum-based chemotherapy, patient background, age, and sex were evaluated. All 23 variables listed above were prespecified as candidate predictors on clinical grounds before modeling, and all are reported in Table 1.

### 2.3. Variable Selection and Nomogram Construction

All 23 prespecified candidate predictors were standardized and entered into a LASSO-penalized logistic regression model [15]. The penalty parameter was selected by 10-fold cross-validation across a grid of 100 values, and the primary model was defined at λ.1se, the largest penalty for which the cross-validated deviance was within one standard error of the minimum. The predictors retained at λ.1se were then refitted without penalization to obtain odds ratios (ORs) and 95% confidence intervals (CIs) for the nomogram. The model selected at λ.min, the penalty that minimized the cross-validated deviance, was fitted as a secondary model for comparison.

The regression coefficients of the selected risk factors were then used to construct a nomogram in which the factor with the greatest contribution was assigned 100 points and the remaining factors were assigned scores proportionally. The total score was subsequently mapped to the predicted probability of HFH using the logistic function.

### 2.4. Model Evaluation and Risk-Stratification Analysis

The primary model was developed using the entire cohort: cross-validated LASSO selection, unpenalized refitting of the retained predictors, nomogram construction, and calculation of predicted probabilities were all performed using the full dataset, and internal validation was conducted by bootstrapping, which is recommended for models with few events [19,20]. A single random split provided an additional held-out estimate. Patients were allocated to the training and test sets in an 8:2 ratio using simple random sampling stratified by outcome, with a fixed random seed. The entire modeling procedure, including cross-validated LASSO selection and coefficient estimation, was repeated using the training set alone, and the resulting model was applied unchanged to the test set.

Discrimination was assessed using the concordance index (C-index), which is equivalent to the area under the receiver operating characteristic curve (AUC), and internal validation was performed using the optimism-corrected C-index and the shrinkage coefficient derived from bootstrap optimism correction (B = 500). Following Harrell’s procedure [21], the complete modeling procedure, including cross-validated LASSO selection, was repeated in each resample so that the uncertainty introduced by variable selection was incorporated into the optimism estimate; the model developed in each resample was evaluated in both that resample and the original cohort, and the mean difference was subtracted from the apparent value. Ninety-five percent confidence intervals were obtained for every performance measure. For the apparent C-index, the Brier score, and the held-out test C-index, they were obtained from 2000 patient-level bootstrap resamples of the fitted predictions. For the optimism-corrected calibration slope and calibration-in-the-large, the intervals were the percentile intervals of the values obtained in the original cohort by the models built in each resample. For the optimism-corrected C-index, the location-shifted bootstrap interval, that is, the apparent interval shifted by the mean optimism, was used [22]. Calibration was assessed using calibration curves and the Hosmer–Lemeshow test [23]. The apparent and optimism-corrected calibration slope and calibration-in-the-large were also reported [24]. The minimum sample size required for a model of this size was calculated according to the criteria of Riley et al. [19]. Decision curve analysis (DCA) was used to quantify the net benefit across a range of threshold probabilities, thereby evaluating the clinical utility of the model.

In addition, the entire cohort was stratified into deciles according to the nomogram-predicted probabilities to compare the incidence of HFH across groups. Cumulative incidence functions were also compared between patients in the highest and lowest 20% of predicted risk and across quartiles of predicted risk.

### 2.5. Time-to-Event and Competing-Risk Analyses

Because the time at risk varied among patients, the logistic model was complemented by time-to-event analyses using the same set of predictors. Time was measured from the index date to HFH, death, or censoring at treatment change, 30 days after the last prescription date, or the end of the observable period. The cumulative incidence of HFH was estimated using the Aalen–Johansen estimator, which treats death as a competing event, and was compared with one minus the Kaplan–Meier estimate to quantify the upward bias associated with the latter [25]. Ninety-five percent confidence intervals were obtained from 1000 patient-level bootstrap resamples. A Fine–Gray subdistribution hazard model was fitted using the LASSO-selected predictors, with death treated as the competing event [26], and a cause-specific Cox proportional hazards model was fitted as a sensitivity analysis. Confidence intervals for the subdistribution hazard ratios were obtained from 1000 patient-level bootstrap resamples rather than from the model-based robust variance, because the bootstrap does not rely on a large-sample normal approximation, which may be less accurate for predictors with few exposed patients [27]; the same resampling approach was used for the cumulative incidence functions. The proportional hazards assumption was examined using the Grambsch–Therneau test based on scaled Schoenfeld residuals [28], and the Cox model was refitted separately for the periods before and after 12 months. A secondary analysis repeated these estimates over the entire observable period. The Aalen–Johansen estimator, Fine–Gray weighted partial likelihood, and Grambsch–Therneau test were implemented by the authors and verified against the corresponding lifelines routines.

### 2.6. Sensitivity Analyses

To distinguish incident from recurrent events, the model was refitted among patients with no HFH before the index date. To examine whether beta-blocker use contributed independently of the conditions for which it was prescribed, the model was also refitted among patients with neither prior HFH nor atrial fibrillation/other arrhythmias (Table S2).

### 2.7. Statistical Analysis

Continuous variables are presented as the mean ± standard deviation or the median (interquartile range [IQR]). Between-group differences were assessed using Student’s *t*-test or the Mann–Whitney U test. Categorical variables are presented as absolute numbers (proportions), and differences were analyzed using the χ^2^ test or Fisher’s exact test. A two-sided *p* value < 0.05 was considered statistically significant. The *p* values in Table 1 are descriptive and were not corrected for multiplicity. Data extraction, merging, and other preprocessing procedures were performed using the KNIME Analytics Platform (version 5.3.3; KNIME AG, Zurich, Switzerland). All statistical analyses were performed using R (version 4.6.1; R Foundation for Statistical Computing, Vienna, Austria) and Python (version 3.13.4; Python Software Foundation, Wilmington, DE, USA) with pandas (version 2.3.1), NumPy (version 2.3.1), scikit-learn (version 1.7.0), statsmodels (version 0.14.6), lifelines (version 0.30.1), and Matplotlib (version 3.10.3). The analysis code is available from the corresponding author upon request.

## 3. Results

### 3.1. Patient Characteristics

Of the 8101 patients with at least one osimertinib prescription, 2 without a lung cancer diagnosis and 2297 with a look-back period of less than six months were excluded; no patient was younger than 18 years, leaving 5802 eligible patients (Figure S2). Among these patients, 169 (2.9%) developed HFH during follow-up. Compared with the non-HFH group, the HFH group was older and had a higher prevalence of cardiovascular risk factors and conditions, including prior HFH, hypertension, ischemic heart disease, atrial fibrillation/other arrhythmias, and valvular disease (Table 1). The median age was 77 years (range, 30–102 years), 34.3% of the patients were men, and atrial fibrillation alone was recorded in 8.9%. The median on-treatment follow-up duration was 9.3 months (IQR, 3.2–20.4 months), corresponding to 6667.9 person-years and an HFH incidence rate of 2.53 per 100 person-years. Follow-up ended because of treatment discontinuation in 3860 patients (66.5%), the end of the available data period in 1505 (25.9%) (the end of March 2023 or loss of insurance eligibility for reasons other than death), death in 268 (4.6%), and HFH in 169 (2.9%); these four categories are mutually exclusive and account for all 5802 patients. The 122 patients who were flagged as deceased but had an uninterpretable eligibility end date (Section 2.1) were not counted as deaths and are included within these categories: their follow-up ended through treatment discontinuation in 111, HFH in 8, and the end of the available data period in 3. Among the 169 events, the median time from the index date to HFH was 153 days (IQR, 44–378 days), and 74% occurred within 12 months. Over the entire observable period, 2358 patients (40.6%) died, and the median overall survival was 31.1 months.

### 3.2. Selected Predictors and the Nomogram

Cross-validated LASSO reached its minimum deviance at λ.min = 0.0022, and the primary model was selected at λ.1se = 0.0054. This model retained seven of the 23 candidate predictors: oral loop diuretic use (largest standardized coefficient, +0.197), age (+0.152), beta-blocker use (+0.133), atrial fibrillation/other arrhythmias (+0.125), tolvaptan use (+0.104), prior HFH (+0.065), and ACE inhibitor use (+0.007) (Table 2, Figure S1). The confidence intervals in Table 2 are conventional post-selection intervals obtained from the refitted model and do not account for the uncertainty introduced by LASSO variable selection; this uncertainty is instead reflected in the bootstrap internal validation, in which the selection step was repeated in every resample (Section 2.4). The secondary model selected at λ.min contained 10 predictors and did not improve discrimination (apparent C-index, 0.729).

Tolvaptan was retained despite its low prevalence of only 0.9% (54 patients, including 13 with events), and its estimate was correspondingly imprecise. Removing tolvaptan reduced the apparent C-index from 0.731 to 0.728.

**Figure 1:**
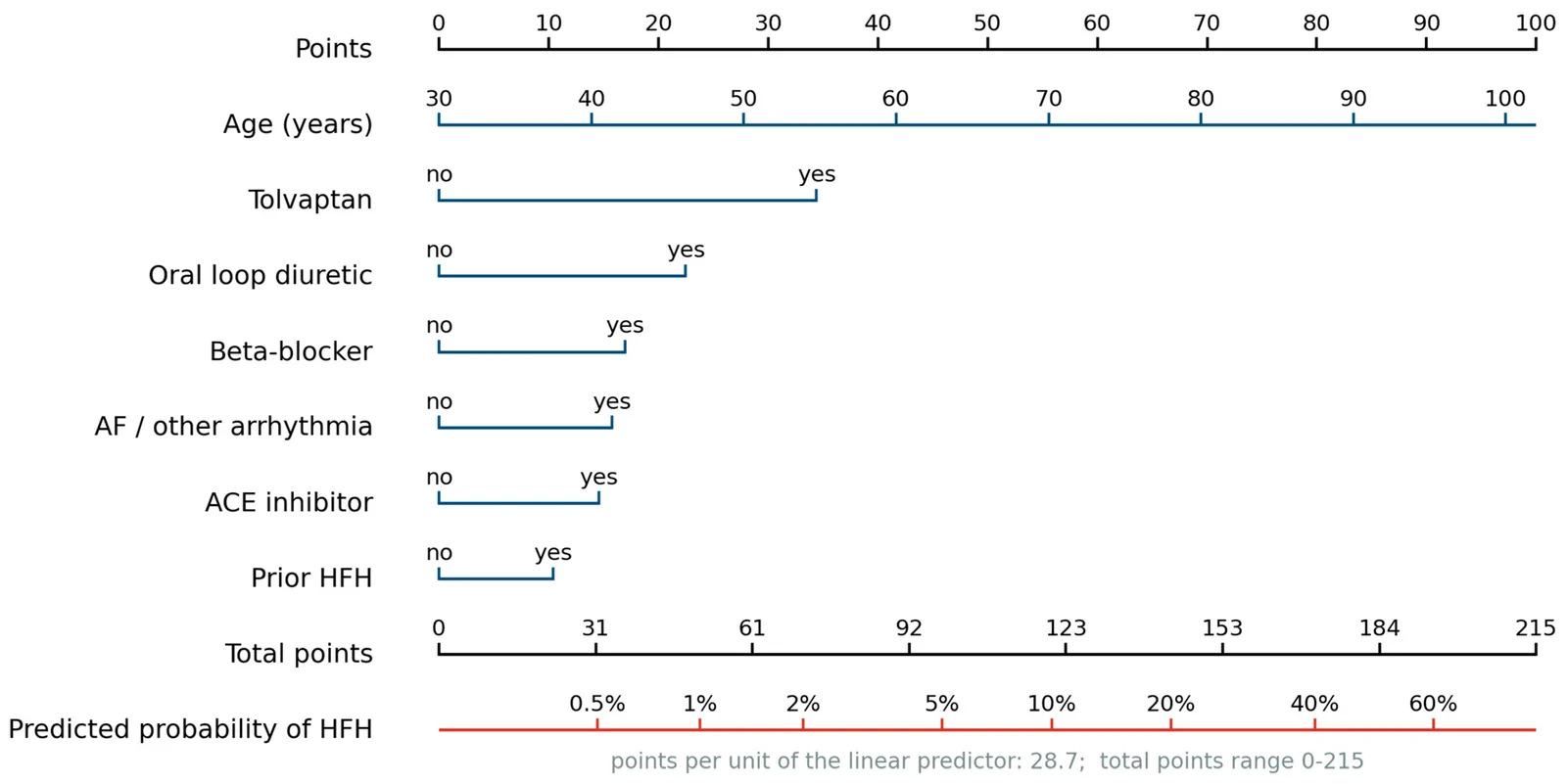
HFH prediction nomogram for patients receiving osimertinib therapy.

The HFH prediction nomogram constructed using these factors is presented in Figure 1. It was designed such that the points assigned to each factor were summed and the total score was then mapped to the corresponding predicted probability of HFH.

The points assigned to each factor are summed to determine the corresponding predicted probability of HFH. For each predictor, the corresponding number of points is read from the uppermost scale. These points are then summed, and the total score is mapped to the predicted probability of HFH on the lowermost scale. Abbreviations: HFH, heart failure hospitalization; AF, atrial fibrillation; ACE, angiotensin-converting enzyme.

### 3.3. Predictive Performance and Internal Validation

**Figure 2:**
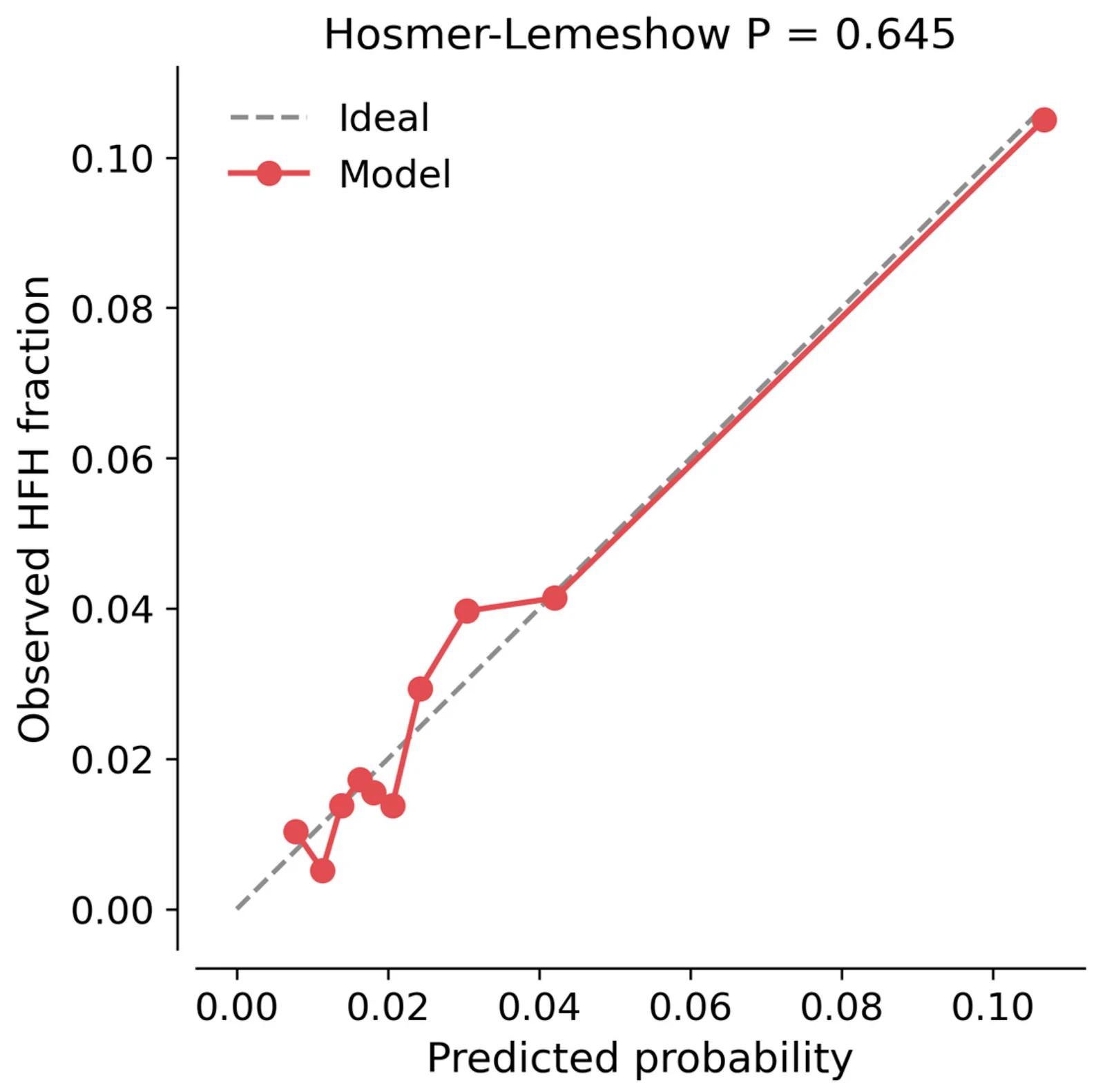
Calibration plot of the primary model.

The predictive performance of the primary and secondary models is summarized in Table 3. The apparent C-index of the primary model was 0.731 (95% CI, 0.691–0.771), and the bootstrap optimism-corrected C-index was 0.718 (95% CI, 0.678–0.757; optimism, 0.013). This estimate was obtained from 499 successful resamples among the 500 in which cross-validated LASSO selection was repeated in full. The optimism-corrected calibration slope was 0.91 (95% CI, 0.51–1.08), and calibration-in-the-large was 0.009 (95% CI, −0.122 to 0.170). Calibration was assessed primarily from the calibration plot (Figure 2), the calibration slope, and calibration-in-the-large, which together suggested reasonable agreement between predicted and observed risks. The Hosmer–Lemeshow test was nonsignificant (χ^2^ = 6.019, degrees of freedom [df] = 8, *p* = 0.645), but a nonsignificant result does not by itself establish good calibration and is reported only as a supplementary check. The axes of the calibration plot span only the range of the decile mean predicted probabilities observed in this cohort, whereas the probability scale of the nomogram (Figure 1) covers the full range of total scores attainable from the predictors; the two figures therefore show different probability ranges. The Brier score was 0.0271 (95% CI, 0.0234–0.0311). According to the criteria of Riley et al. [19], a model with seven predictors, an outcome proportion of 2.91%, and an apparent Cox–Snell R^2^ of 0.024 requires at least 2611 patients to maintain the expected shrinkage below 10%; therefore, the available sample of 5802 patients with 169 events (24.1 events per predictor) exceeded the minimum requirement.

The diagonal dashed line represents perfect calibration. The *x*-axis represents the mean predicted probability in each decile, and the *y*-axis represents the observed proportion of HFH. Points closer to the diagonal (perfect calibration) indicate better calibration. HFH, heart failure hospitalization.

In the single random 8:2 split, the training set comprised 4641 patients, including 135 with HFH (2.91%), and the test set comprised 1161 patients, including 34 with HFH (2.93%). The model redeveloped using the training set retained the same seven predictors and yielded a training C-index of 0.726, whereas the held-out test C-index was 0.753 (95% CI, 0.672–0.830), with a test calibration slope of 0.822. This estimate was based on only 34 events and was therefore correspondingly imprecise.

### 3.4. Decision Curve Analysis

**Figure 3:**
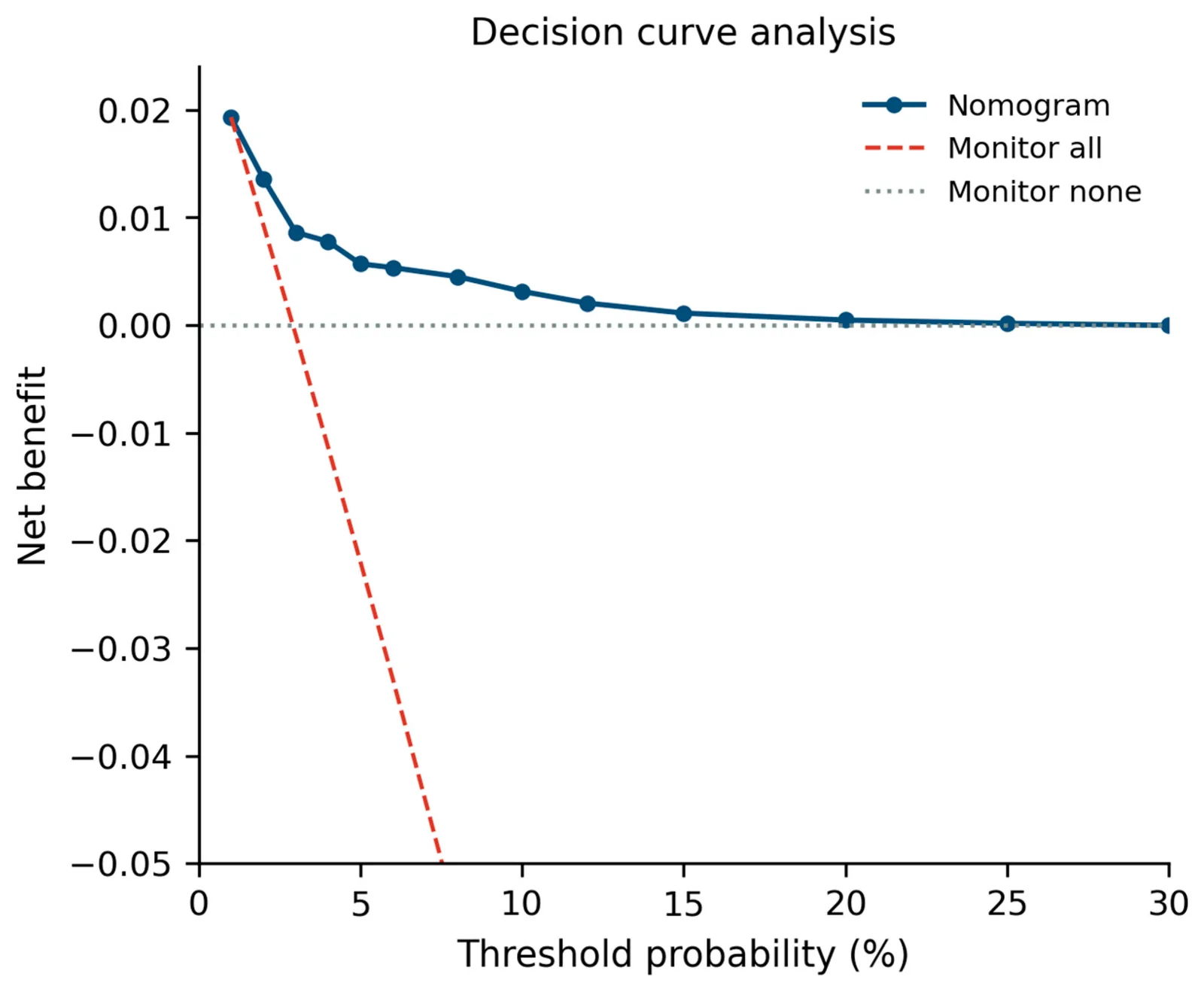
Decision curve analysis of the primary model.

Across a clinically relevant range of low threshold probabilities (approximately 1–25%), the primary model yielded a greater net benefit than both the treat-all and treat-none strategies (Figure 3). However, because the prevalence of HFH was low (2.9%), the absolute net benefit was modest, and no benefit was observed at higher thresholds.

The *x*-axis represents the threshold probability, and the *y*-axis represents the net benefit. Net benefit is calculated by subtracting the loss associated with false-positive results, weighted according to the threshold odds, from the benefit conferred by true-positive results.

### 3.5. Risk-Stratification Analysis

**Figure 4:**
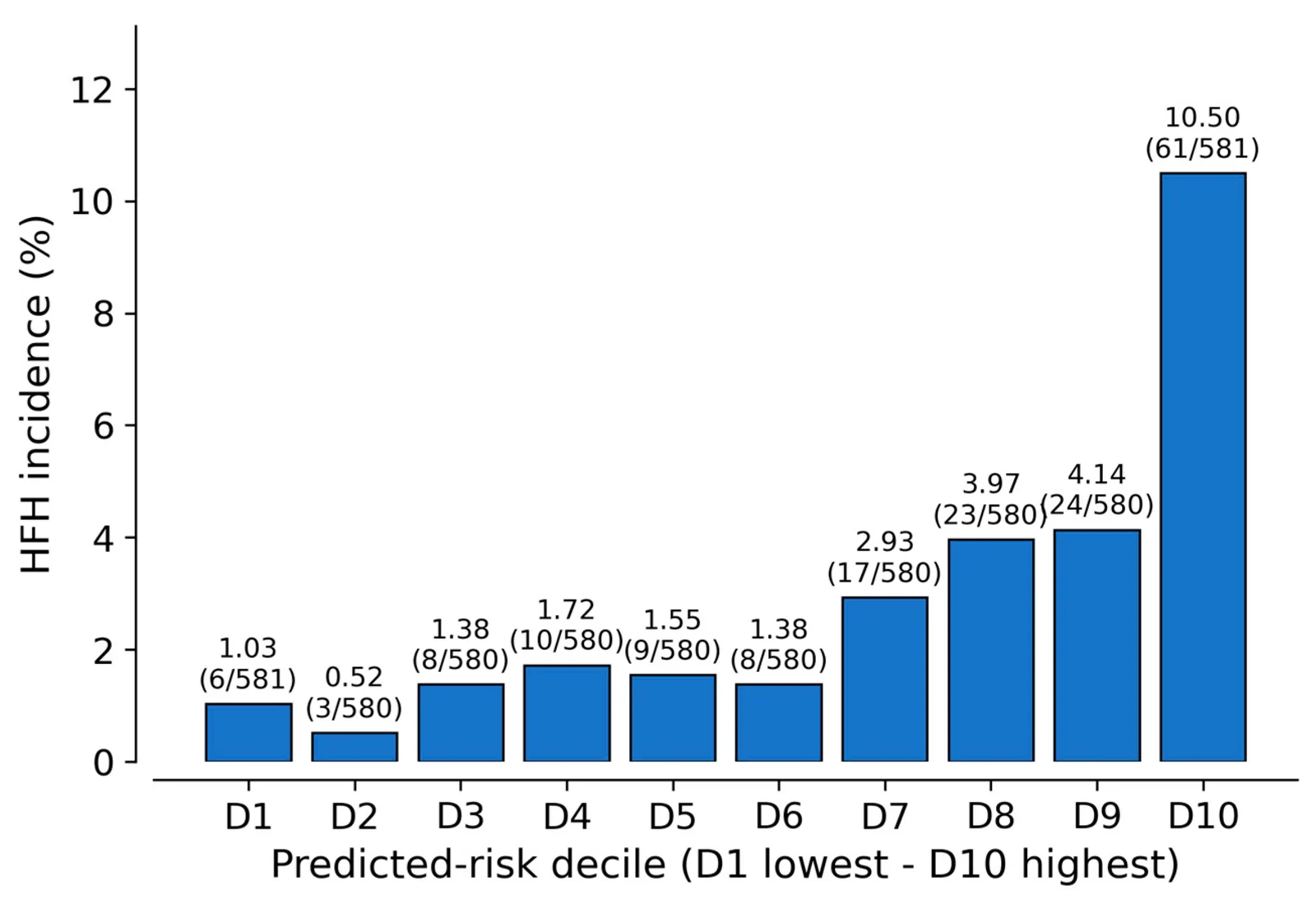
Observed HFH incidence by predicted-risk decile in the primary model.

When the cohort was stratified into deciles according to the probabilities predicted by the primary model, the observed incidence of HFH increased approximately tenfold, from 1.03% in the lowest-risk decile (D1) to 10.50% in the highest-risk decile (D10) (Figure 4); the mean predicted probability closely tracked the observed incidence in each decile.

Using the probabilities predicted by the primary model, the entire cohort was stratified into deciles, ranging from D1 (lowest risk) to D10 (highest risk). The observed incidence of HFH (%) in each decile is presented as a bar chart. The values above the bars indicate the incidence and the numbers of events and patients. HFH, heart failure hospitalization.

**Figure 5:**
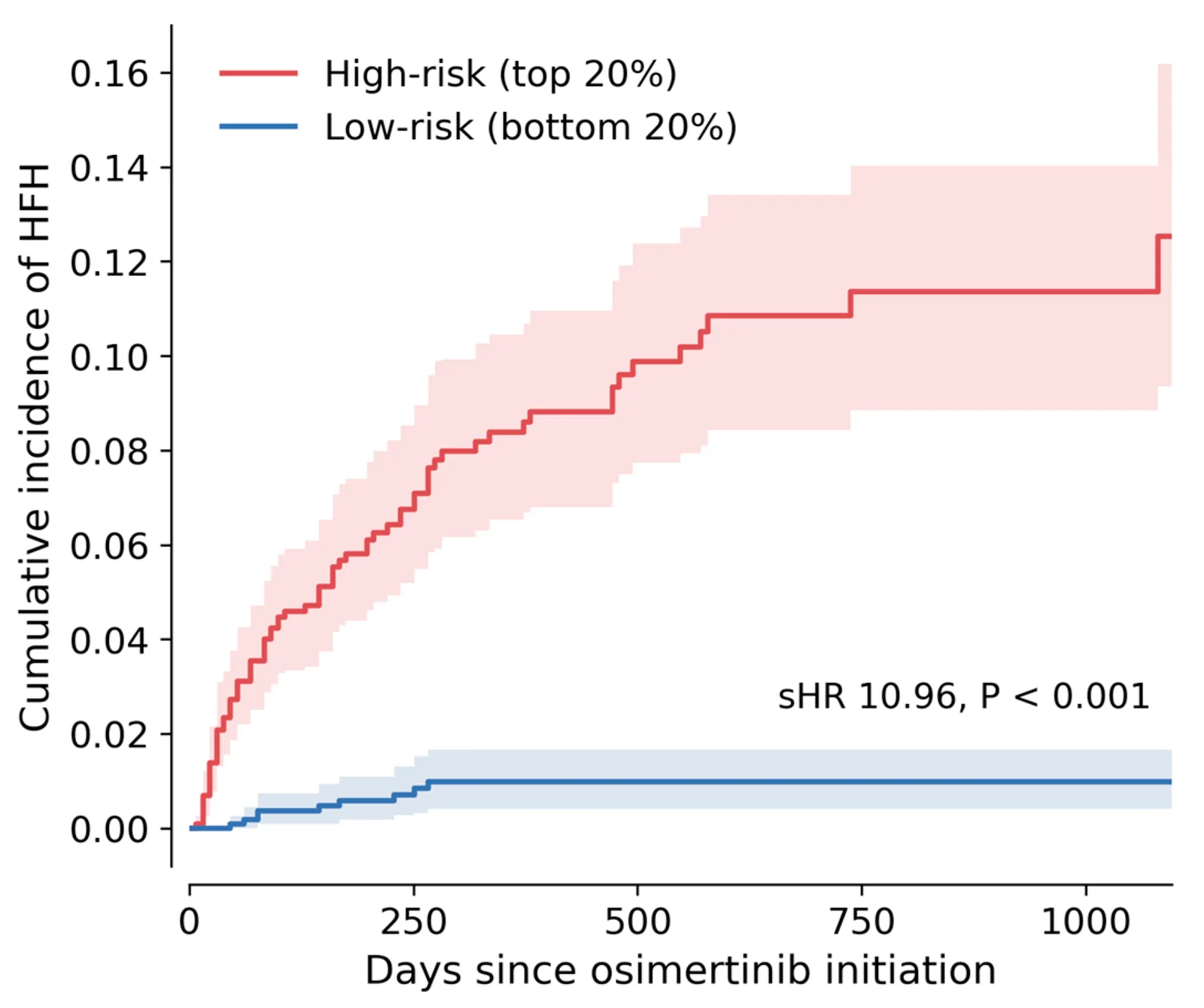
Cumulative incidence of HFH stratified by predicted risk.

In the comparison between the highest and lowest 20% of predicted risk, the high-risk group had a significantly higher incidence of HFH (Figure 5). The 12-month cumulative incidence was 8.38% in the high-risk group and 0.98% in the low-risk group, and the subdistribution hazard ratio was 10.96 (95% CI, 5.68–21.15; *p* < 0.001). Across the quartiles of predicted risk, the 12-month cumulative incidence increased from 1.09% to 6.94%, and the Fine–Gray predictions for the corresponding quartiles (0.87–7.43%) were consistent with the observed values.

The curves depict the cumulative incidence of HFH during follow-up in the high-risk (top 20%) and low-risk (bottom 20%) groups, as defined by the risk predicted by the primary model. The shaded areas represent the 95% confidence intervals. Because death is a competing event, cumulative incidence was estimated using the Aalen–Johansen estimator rather than the Kaplan–Meier method, which would overestimate the probability of HFH. Confidence intervals were obtained from 1000 patient-level bootstrap resamples, and the between-group difference was assessed using the subdistribution hazard ratio from a Fine–Gray model. HFH, heart failure hospitalization; sHR, subdistribution hazard ratio.

### 3.6. Competing-Risk and Cause-Specific Estimates

During the on-treatment period, 169 HFH events and 268 competing deaths occurred, corresponding to a ratio of 1.59 competing deaths per primary event. The Aalen–Johansen cumulative incidence of HFH was 1.26% at 3 months, 2.85% (95% CI, 2.33–3.33) at 12 months, and 5.41% at 36 months. One minus the Kaplan–Meier estimate exceeded these values by 3% at 12 months and 5% at 36 months, indicating that the bias associated with the Kaplan–Meier approach was small within the on-treatment period; over the entire observable period, during which 2155 competing deaths occurred, the same comparison showed overestimations of 17% at 24 months and 28% at 36 months.

For every predictor, the Fine–Gray model yielded subdistribution hazard ratios that were consistent in direction and magnitude with the logistic odds ratios, and the cause-specific Cox model produced closely corresponding estimates (Table 4). The Pearson correlation between the logistic and Fine–Gray estimates on the logarithmic scale was 0.992, as was the correlation between the logistic and Cox estimates. The Harrell C-index [21] of the Cox risk score was 0.771. The Grambsch–Therneau test provided no overall evidence against the proportional hazards assumption (global χ^2^ = 12.20, 7 df, *p* = 0.094); oral loop diuretic use was the only predictor with a nominally significant result (*p* = 0.029), and refitting the Cox model separately for the periods before and after 12 months did not alter the direction of any association.

### 3.7. Incident Events and Beta-Blocker Use

Restricting the analysis to the 5195 patients with no prior HFH, such that only incident events were included, left the direction of every association unchanged and yielded an apparent C-index of 0.687, with a decile gradient ranging from 0.98% to 6.63%. Thus, the model retained discriminatory ability after patients with prior HFH were excluded, although its discrimination was lower than that in the overall cohort.

Among the 4195 patients with neither prior HFH nor atrial fibrillation/other arrhythmias, HFH occurred in 4.47% of beta-blocker users and 1.75% of non-users, and beta-blocker use remained associated with HFH after adjustment (OR, 2.11; 95% CI, 1.08–4.12). Nevertheless, in the whole cohort, beta-blocker users had a substantially greater cardiovascular burden than non-users, including a higher prevalence of prior HFH (33.0% vs. 7.5%), atrial fibrillation/other arrhythmias (50.8% vs. 17.9%), and ischemic heart disease (50.7% vs. 16.7%); these prevalences refer to all 5802 patients and not to the restricted subgroup, in which prior HFH and atrial fibrillation/other arrhythmias are absent by definition (Table S2).

## 4. Discussion

This is the first study to identify baseline factors associated with HFH during osimertinib therapy using penalized regression and to construct and validate a practical nomogram derived from claims data. A salient finding was that the risk of HFH depended on patients’ underlying cardiovascular vulnerability, including advanced age, prior HFH, and atrial fibrillation/other arrhythmias. This finding is broadly consistent with the multivariable analyses of osimertinib-related cardiotoxicity reported by Bak et al. [12] and heart failure reported by Tatebe et al. [29]. The emergence of beta-blocker use as a predictor in the present study was considered to reflect its use as a treatment for underlying cardiovascular conditions rather than a risk associated with the drug itself. The sensitivity analyses support this interpretation: in the whole cohort, beta-blocker users had two- to threefold higher prevalences of prior HFH, arrhythmias, and ischemic heart disease; therefore, this variable is best understood as an aggregate marker of cardiovascular disease burden. The same interpretation applies to the other medication-related predictors: ACE inhibitors indicate treated hypertension, chronic kidney disease, or heart failure, whereas tolvaptan and oral loop diuretics are prescribed to manage congestion and therefore identify patients with established heart failure. None of these estimates should be interpreted as causal, and none suggest that discontinuing the medication would reduce risk.

The constructed nomogram showed moderate discrimination (optimism-corrected C-index, 0.718) and good calibration (optimism-corrected slope, 0.91; 95% CI, 0.51–1.08). The moderate discrimination reflects the difficulty of predicting a multifactorial event such as HFH using claims-based variables alone. The strength of the model is that it uses factors ascertainable for every patient, namely diagnoses, age, and concomitant medications, to identify populations for whom cardiovascular evaluation should be intensified. In the stratification analysis, an approximately tenfold gradient in incidence was observed across the predicted-risk deciles, and the DCA demonstrated a net benefit, although this benefit was observed only at low threshold probabilities and was small in absolute terms because HFH is rare. At a threshold of 5%, the net benefit of 0.0057 corresponds to the identification of approximately six additional patients with HFH per 1000 patients assessed, after false-positive results have been subtracted at the weight implied by that threshold. Because the burden and harms of additional cardiovascular testing were not evaluated empirically, this value should be read as a model-based summary of the decision curve rather than as evidence of clinical benefit. The model is therefore best regarded as an auxiliary screening tool for identifying subgroups that warrant intensified cardiovascular monitoring rather than as an instrument for quantifying individual risk. In practice, a patient in the highest decile, whose predicted risk exceeds 5%, would be a candidate for baseline echocardiography and natriuretic peptide measurement, review of diuretic and rate-control therapy, and, where available, cardio-oncology referral before osimertinib initiation. Symptom-directed reassessment would then follow during the first year, when three-quarters of the events occurred. The Heart Failure Association–International Cardio-Oncology Society (HFA-ICOS) baseline risk-stratification tool is used for pretreatment risk assessment and is recommended in the 2022 European Society of Cardiology (ESC) cardio-oncology guidelines [30,31]. It includes HER2 inhibitors among its drug classes but does not encompass agents with distinctive cardiovascular mechanisms, such as osimertinib. The present nomogram is intended to address this gap among patients starting osimertinib, and its predictors, including age, prior HFH, and atrial fibrillation, overlap with the high-risk parameters of HFA-ICOS. Because all patients in this cohort received osimertinib and no comparator group was available, we could not determine whether these factors indicate susceptibility specifically to osimertinib-associated cardiotoxicity or a general susceptibility to HFH. Furthermore, the discrimination of the nomogram was within the same moderate range as that of the HFA-ICOS score in its external validation for anthracycline-related cardiovascular toxicity, in which the AUC and Uno’s C-statistic were both 0.78 at 12 months [32].

The median on-treatment follow-up duration was 9.3 months, and the time at risk varied widely among patients. Logistic regression was retained as the primary model because the prespecified objective was to develop a simple baseline screening tool for determining whether HFH would occur during the observed osimertinib treatment period. We acknowledge that this estimand depends partly on treatment duration and does not represent risk over a fixed time horizon. We therefore complemented the primary analysis with competing-risk and cause-specific time-to-event analyses. The estimates from the Fine–Gray and cause-specific Cox models were closely aligned with those from the logistic model (correlation, 0.992 on the logarithmic scale), supporting the robustness of the identified predictor associations. Death was a genuine competing event: 268 patients died during treatment, and 2358 (40.6%) died over the entire observable period, with a median overall survival of 31.1 months. Within the on-treatment period, the Kaplan–Meier approach overestimated the cumulative incidence of HFH by only 3–5%; however, over the entire observable period, the overestimation reached 28% at 36 months. Therefore, cumulative incidence functions are reported throughout.

Records with a suspected-diagnosis flag, which is entered in Japanese claims to justify diagnostic testing, were excluded from the definition of every comorbidity. Including these records would have approximately doubled the recorded prevalences of diabetes mellitus, ischemic heart disease, atrial fibrillation/other arrhythmias, and valvular disease, whereas the prevalences reported here approximate published estimates for older Japanese patients with advanced NSCLC. Because cross-validated LASSO selection was repeated within every bootstrap resample, the estimated optimism (0.013) also incorporates the uncertainty introduced by variable selection.

Several approaches could improve the model. First, the outcome was restricted to severe HFH; consequently, the incidence was low (2.9%), and the limited number of events constrained both discrimination and positive predictive value. Osimertinib-related cardiac dysfunction is reported considerably more frequently when milder manifestations, such as a reduction in left ventricular ejection fraction or outpatient-managed heart failure, are included [8,12,14,33]. A composite outcome would therefore increase the effective number of events and might yield a more stable model. Second, cardiovascular information not captured in claims data, such as echocardiographic findings, B-type natriuretic peptide (BNP)/N-terminal pro-BNP (NT-proBNP) levels, electrocardiographic findings, and longitudinal changes in laboratory values or body weight, would better reflect subclinical cardiac dysfunction. Third, model development in multicenter cohorts, followed by temporal and external validation, would allow its generalizability to be assessed.

Cardiac biomarkers and imaging data were not available. Natriuretic peptides and left ventricular ejection fraction are established predictors of heart failure, and their absence may partly explain the moderate discrimination of the present claims-based model. However, because these variables were unavailable in the database, we could not determine the direction or magnitude of their effects on individual predictor coefficients or quantify the extent to which model performance would improve if they were included. The nomogram should therefore be used only when biomarker and imaging data are unavailable, as a first-pass screening tool for identifying patients who may warrant further cardiovascular assessment, and never as a substitute for biomarker or imaging evaluation.

This study has some limitations. First, because of its retrospective, real-world design, residual confounding cannot be entirely excluded. Because this was a single-arm cohort in which every patient received osimertinib, no inferences could be made regarding cardiotoxicity attributable to osimertinib, and the associations reported here are predictive rather than causal. Second, claims data lack detailed cardiovascular information, such as left ventricular ejection fraction, BNP/NT-proBNP levels, and electrocardiographic findings. Third, restricting HFH to events requiring hospitalization resulted in a low event count. Because the outcome definition required intravenous decongestive therapy, patients already receiving oral loop diuretics or tolvaptan may have been more likely to meet this definition once admitted; therefore, the coefficients of these two predictors may have been inflated. Fourth, the HFH algorithm has not been validated against medical records from this database, although it follows an algorithm validated using other Japanese claims data. In addition, censoring due to loss of insurance eligibility or a change of insurer may not be independent of the outcome, so informative censoring cannot be excluded. Fifth, the composite category of atrial fibrillation and other arrhythmias is clinically heterogeneous, and the sparse rhythm-specific codes precluded separate analyses. Sixth, transportability is limited: the cohort had a median age of 77 years, was derived from a single Japanese insurer-based database, and reflects Japanese prescribing patterns; therefore, both the coefficients and baseline risk will likely require recalibration in other settings. Finally, the model was not externally validated in the present study. The nomogram is a simple tool for identifying patients who warrant regular cardiovascular evaluation, and its clinical value should be considered provisional pending external validation.

## 5. Conclusions

HFH during osimertinib therapy was associated with baseline factors reflecting cardiovascular vulnerability, namely age; prior HFH; atrial fibrillation/other arrhythmias; and the use of beta-blockers, ACE inhibitors, oral loop diuretics, and tolvaptan. The nomogram constructed using these factors showed moderate discrimination and good calibration and separated the cohort into strata with an approximately tenfold difference in observed incidence. It may therefore be more useful as an adjunctive screening aid for identifying patients who require pretreatment cardiovascular assessment and intensified monitoring than as a tool for predicting individual risk. External validation and refinement of the nomogram using biomarker and imaging data are required before it can be used more widely.

## Figures and Tables

**Table 1:** Baseline characteristics of patients receiving osimertinib.

Characteristic	Overall (*N* = 5802)	No HFH (*n* = 5633)	HFH (*n* = 169)	*p* Value
Age, median (min-max), years	77 (30–102)	77 (30–102)	80 (51–96)	<0.001
Male sex, *n* (%)	1991 (34.3%)	1932 (34.3%)	59 (34.9%)	0.869
Prior HFH, *n* (%)				
No prior HFH	5195 (89.5%)	5073 (90.1%)	122 (72.2%)	<0.001
Prior HFH	607 (10.5%)	560 (9.9%)	47 (27.8%)	
Comorbidities, *n* (%)				
Hypertension	3899 (67.2%)	3763 (66.8%)	136 (80.5%)	<0.001
Diabetes	2380 (41.0%)	2299 (40.8%)	81 (47.9%)	0.064
Dyslipidemia	3131 (54.0%)	3030 (53.8%)	101 (59.8%)	0.125
Ischemic heart disease	1198 (20.6%)	1140 (20.2%)	58 (34.3%)	<0.001
AF/arrhythmia	1259 (21.7%)	1186 (21.1%)	73 (43.2%)	<0.001
Cardiomyopathy	53 (0.9%)	50 (0.9%)	3 (1.8%)	0.200
Valvular disease	436 (7.5%)	408 (7.2%)	28 (16.6%)	<0.001
CKD	350 (6.0%)	331 (5.9%)	19 (11.2%)	0.004
COPD	2125 (36.6%)	2062 (36.6%)	63 (37.3%)	0.858
Alcohol-related disorder	8 (0.1%)	8 (0.1%)	0 (0.0%)	1.000
Alcoholic liver disease	48 (0.8%)	47 (0.8%)	1 (0.6%)	1.000
Cardiovascular medications, *n* (%)				
Beta-blocker	669 (11.5%)	617 (11.0%)	52 (30.8%)	<0.001
ACE inhibitor	153 (2.6%)	140 (2.5%)	13 (7.7%)	<0.001
ARB	2001 (34.5%)	1934 (34.3%)	67 (39.6%)	0.152
MRA	217 (3.7%)	198 (3.5%)	19 (11.2%)	<0.001
SGLT2 inhibitor	126 (2.2%)	123 (2.2%)	3 (1.8%)	1.000
Loop diuretic (oral)	598 (10.3%)	543 (9.6%)	55 (32.5%)	<0.001
Tolvaptan	54 (0.9%)	41 (0.7%)	13 (7.7%)	<0.001
Concomitant chemotherapy, *n* (%)				
Cisplatin	74 (1.3%)	72 (1.3%)	2 (1.2%)	1.000
Carboplatin	213 (3.7%)	209 (3.7%)	4 (2.4%)	0.530

HFH, heart failure hospitalization; AF, atrial fibrillation; CKD, chronic kidney disease; COPD, chronic obstructive pulmonary disease; ACE, angiotensin-converting enzyme; ARB, angiotensin II receptor blocker; MRA, mineralocorticoid receptor antagonist; SGLT2, sodium–glucose cotransporter 2. Diagnosis records flagged as suspected were excluded (Section 2.2). Continuous variables were compared with the Mann–Whitney U test and proportions with the χ^2^ test, or Fisher’s exact test when any expected count was below 5; *p* values are descriptive.

**Table 2:** Multivariable logistic regression on the LASSO-selected predictors.

Variable	Adjusted OR (95% CI)	*p* Value
Age	1.050 (1.026–1.074)	<0.001
Beta-blocker use	1.806 (1.219–2.675)	0.003
AF/arrhythmia	1.732 (1.228–2.442)	0.002
Prior HFH	1.435 (0.949–2.169)	0.087
ACE inhibitor use	1.660 (0.870–3.168)	0.124
Loop diuretic use (oral)	2.189 (1.471–3.257)	<0.001
Tolvaptan use	3.316 (1.586–6.937)	0.001

Predictors retained by 10-fold cross-validated least absolute shrinkage and selection operator (LASSO) regression at λ.1se (the largest penalty within one standard error of the minimum cross-validated deviance) and refitted without penalty. The confidence intervals are conventional post-selection intervals and do not account for the uncertainty introduced by LASSO variable selection (Section 3.2). OR, odds ratio; CI, confidence interval; HFH, heart failure hospitalization; AF, atrial fibrillation; ACE, angiotensin-converting enzyme.

**Table 3:** Predictive performance of the primary and secondary models.

Measure	Primary Model (7 Predictors)	Secondary Model (10 Predictors, λ.min)
Apparent C-index	0.731 (0.691–0.771)	0.729 (0.687–0.768)
Optimism-corrected C-index	0.718 (0.678–0.757)	0.719 (0.677–0.759)
C-index, held-out test set	0.753 (0.672–0.830)	0.742 (0.649–0.827)
Optimism-corrected calibration slope	0.91 (0.51–1.08)	–
Optimism-corrected calibration-in-the-large	0.009 (−0.122 to 0.170)	–
Brier score	0.0271 (0.0234–0.0311)	–
Hosmer–Lemeshow *p*	0.645	0.920

Values are estimates with 95% confidence intervals (Section 2.4). For the main model, the optimism-corrected C-index and the calibration slope derive from Harrell’s bootstrap (B = 500), in which the cross-validated least absolute shrinkage and selection operator (LASSO) selection was repeated in every resample; for the secondary model, the predictor set was held fixed and 500 resamples were used. The held-out test C-index was calculated using an 80/20 training/test split, allocated by simple random sampling stratified on the outcome with a fixed random seed. A dash indicates a measure that was not computed for the secondary model. C-index, concordance index; λ.min, the penalty that minimized the cross-validated deviance.

**Table 4:** Comparison of the logistic, Fine–Gray, and cause-specific Cox models on the identical predictor set.

Variable	OR (Logistic)	sHR (Fine–Gray)	95% CI (sHR)	HR (Cox)
Age	1.050	1.057	1.033–1.085	1.061
Beta-blocker use	1.806	1.843	1.193–2.781	1.766
AF/arrhythmia	1.732	1.839	1.293–2.590	1.877
Prior HFH	1.435	1.533	0.990–2.258	1.551
ACE inhibitor use	1.660	1.920	0.993–3.354	1.850
Loop diuretic use (oral)	2.189	2.323	1.463–3.461	2.435
Tolvaptan use	3.316	3.405	1.625–6.844	3.534

OR, odds ratio; sHR, subdistribution hazard ratio; CI, confidence interval; HR, hazard ratio; HFH, heart failure hospitalization; AF, atrial fibrillation; ACE, angiotensin-converting enzyme. Confidence intervals for the subdistribution hazard ratios are from 1000 patient-level bootstrap resamples. Death is the competing event in the Fine–Gray model and a censoring event in the cause-specific Cox model.

## Data Availability

The data supporting the findings of this study were obtained from the DeSC database (DeSC Healthcare, Inc., Tokyo, Japan) under a data-use agreement and are therefore not publicly available. Restrictions apply to the availability of these data, which were used under license for the current study. The data may be made available by DeSC Healthcare, Inc. upon reasonable request and with permission from DeSC Healthcare, Inc.

## Supplementary Materials

The following supporting information can be downloaded at: https://www.mdpi.com/article/10.3390/curroncol33100586/s1. Table S1: TRIPOD checklist for the development and internal validation of a prediction model; Table S2: Subgroup analyses (incident HFH and beta-blocker use); Figure S1: Cross-validated LASSO for the primary model; Figure S2: Flow of participants.

## Abbreviations

The following abbreviations are used in this manuscript:

ACE	angiotensin-converting enzyme
AF	atrial fibrillation
ARB	angiotensin II receptor blocker
AUC	area under the receiver operating characteristic curve
BNP	B-type natriuretic peptide
C-index	concordance index
CI	confidence interval
CKD	chronic kidney disease
COPD	chronic obstructive pulmonary disease
DCA	decision curve analysis
df	degrees of freedom
EGFR	epidermal growth factor receptor
ESC	European Society of Cardiology
HER2	human epidermal growth factor receptor 2
HFA-ICOS	Heart Failure Association–International Cardio-Oncology Society
HFH	heart failure hospitalization
HR	hazard ratio
ICD-10	International Classification of Diseases, 10th Revision
IQR	interquartile range
LASSO	least absolute shrinkage and selection operator
MRA	mineralocorticoid receptor antagonist
NSCLC	non-small cell lung cancer
NT-proBNP	N-terminal pro-B-type natriuretic peptide
OR	odds ratio
SGLT2	sodium–glucose cotransporter 2
sHR	subdistribution hazard ratio
TKI	tyrosine kinase inhibitor
TRIPOD	Transparent Reporting of a multivariable prediction model for Individual Prognosis Or Diagnosis
