# Supplementary material for: Risk Stratification and Factors Associated with Heart Failure Hospitalization During Osimertinib Therapy
Source: Curr Oncol. 2026 Sep 29;33(10):586. doi: 10.3390/curroncol33100586 (PMC13703609; doi:10.3390/curroncol33100586)
Supplement: Supplementary file 1 [file curroncol-33-00586-s001.zip › curroncol-4485037-supplementary.pdf]

Supplementary Materials

Table S1. TRIPOD checklist for the development and internal validation of a prediction model

Item	Section	Checklist item	Where addressed
1	Title	Title identifies the study as developing and internally validating a prediction model, names the outcome and the population	Title
2	Abstract	Structured summary of objectives, setting, participants, outcome, predictors, sample size, statistical analysis, results and conclusions	Abstract
3a	Background	Rationale, including a reference to existing models	Introduction; Discussion
3b	Objectives	Objectives stated, including whether development or validation	Introduction, final paragraph
4a	Source of data	Study design and data source described	Section 2.1
4b	Source of data	Key dates of data accrual and follow-up	Sections 2.1, 2.2, 3.1
5a	Participants	Setting and eligibility criteria	Section 2.1
5b	Participants	Details of treatment received	Sections 2.1, 2.2 (on-treatment window)
6a	Outcome	Outcome definition and how and when it was measured	Section 2.2
6b	Outcome	Blinding of outcome assessment; actions to avoid outcome ascertainment bias	Section 2.2
7a	Predictors	All predictors defined, including how and when measured	Section 2.2
7b	Predictors	Predictor assessment blinded to outcome	Section 2.2: all predictors precede the index date
8	Sample size	How the study size was arrived at	Sections 2.4, 3.3 (Riley criteria)
9	Missing data	How missing data were handled	Section 2.1 (records with an uninterpretable eligibility-end value censored)
10a	Statistical analysis	How predictors were handled in the analysis	Sections 2.2, 2.3
10b	Statistical analysis	Model type, model-building procedures and internal validation	Sections 2.3, 2.4, 2.5
10d	Statistical analysis	Measures of model performance	Section 2.4
10e	Statistical analysis	Any model updating	None performed
11	Risk groups	How risk groups were created	Sections 2.4, 3.5
13a	Participants	Flow of participants, with numbers at each stage	Section 3.1; Figure S2
13b	Participants	Characteristics of participants, including predictors and outcome	Table 1
14a	Model development	Number of participants and outcome events	Sections 3.1, 3.3
14b	Model development	Unadjusted association between each predictor and the outcome	Table 1
15a	Model specification	Full prediction model, including all regression coefficients and the intercept	Table 2, Figure 1
15b	Model specification	How to use the model	Figure 1; Discussion
16	Model performance	Performance measures with confidence intervals	Table 3, Figure 2
17	Model updating	Results of any model updating	Not applicable
18	Limitations	Limitations, including non-representativeness and missing data	Discussion, final paragraphs

19b	Interpretation	Overall interpretation, with reference to performance and other evidence	Discussion
20	Implications	Potential clinical use and implications for future research	Discussion; Conclusions
21	Supplementary	Information about supplementary resources	Supplementary Tables S1-S2, Figures S1-S2
22	Funding	Role of the funders	Funding statement

Items 12 and 19a apply only to validation studies and are therefore not applicable.

Table S2. Subgroup analyses (incident HFH and beta-blocker use)

Analysis	OR / value	95% CI	P value
**Patients without prior HFH**			
n / HFH events	5195 / 122		
Age, years	1.051	1.024-1.078	<0.001
Beta-blocker	2.025	1.255-3.267	0.004
ACE inhibitor	1.251	0.488-3.210	0.641
Oral loop diuretic	2.244	1.372-3.672	0.001
Tolvaptan	3.164	0.831-12.042	0.091
Atrial fibrillation / other arrhythmia	1.615	1.078-2.418	0.020
C-index	0.687		
Incidence, decile 1 to decile 10	0.98% to 6.63%		
**Patients without prior HFH and without arrhythmia**			
n / HFH events	4195 / 80		
HFH in beta-blocker users	4.47%		
HFH in non-users	1.75%		
Age, years	1.060	1.027-1.094	<0.001
Beta-blocker	2.112	1.082-4.121	0.028
ACE inhibitor	0.820	0.188-3.569	0.791
Oral loop diuretic	2.710	1.435-5.115	0.002
Tolvaptan	2.830	0.550-14.573	0.213
**Cardiovascular burden of beta-blocker users (whole cohort)**	Users (%)	Non-users (%)	
Prior HFH	33.0	7.5	
Atrial fibrillation / other arrhythmia	50.8	17.9	
Ischemic heart disease	50.7	16.7	
Arterial hypertension	95.1	63.6	
Valvular heart disease (non-rheumatic)	17.5	6.2	
Cardiomyopathy	4.5	0.4	
Chronic kidney disease	13.5	5.1	
Oral loop diuretic	28.4	7.9	

The first subgroup contains only patients who had never been hospitalized for heart failure, so every event is an incident HFH. In the last block the second and third columns give the prevalence of each condition among beta-blocker users and non-users in the whole cohort of 5,802 patients.

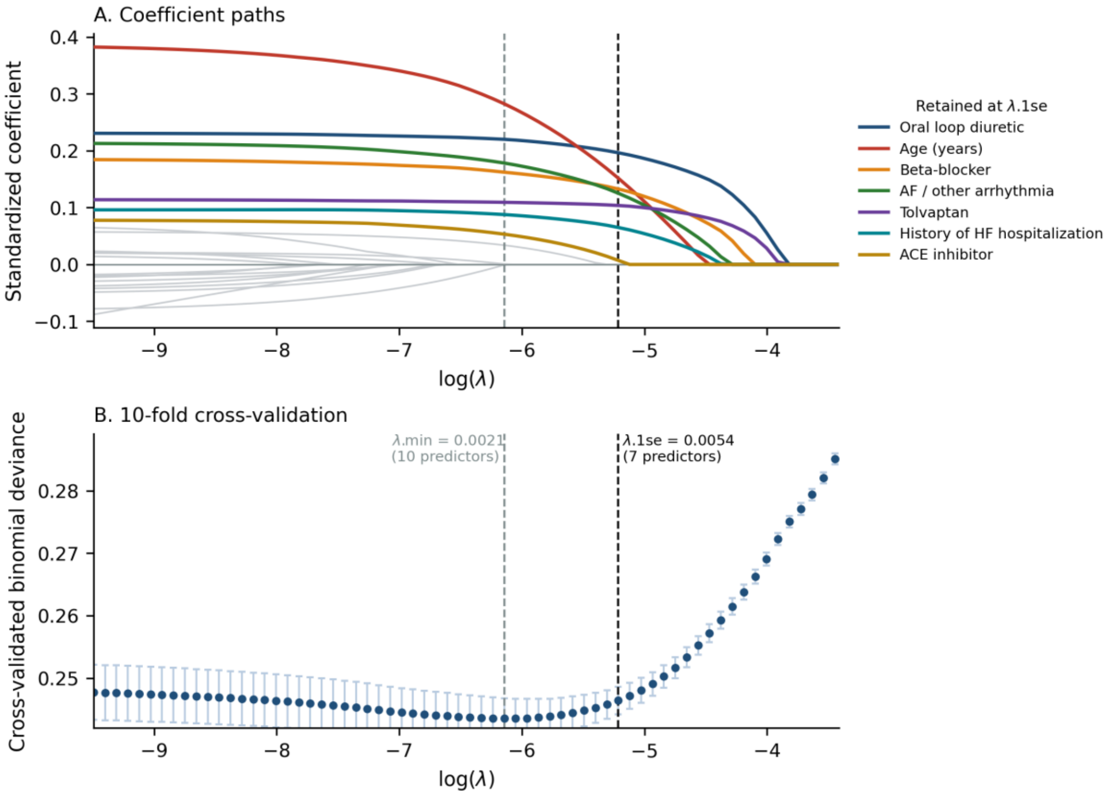

Figure S1. Cross-validated LASSO for the primary model. (A) Standardized coefficient paths against log(λ); the predictors retained at λ.1se are drawn in colour and the remaining candidates in grey. (B) Ten-fold cross-validated binomial deviance against log(λ); the dashed lines mark λ.min and λ.1se, and the primary model was taken at λ.1se.

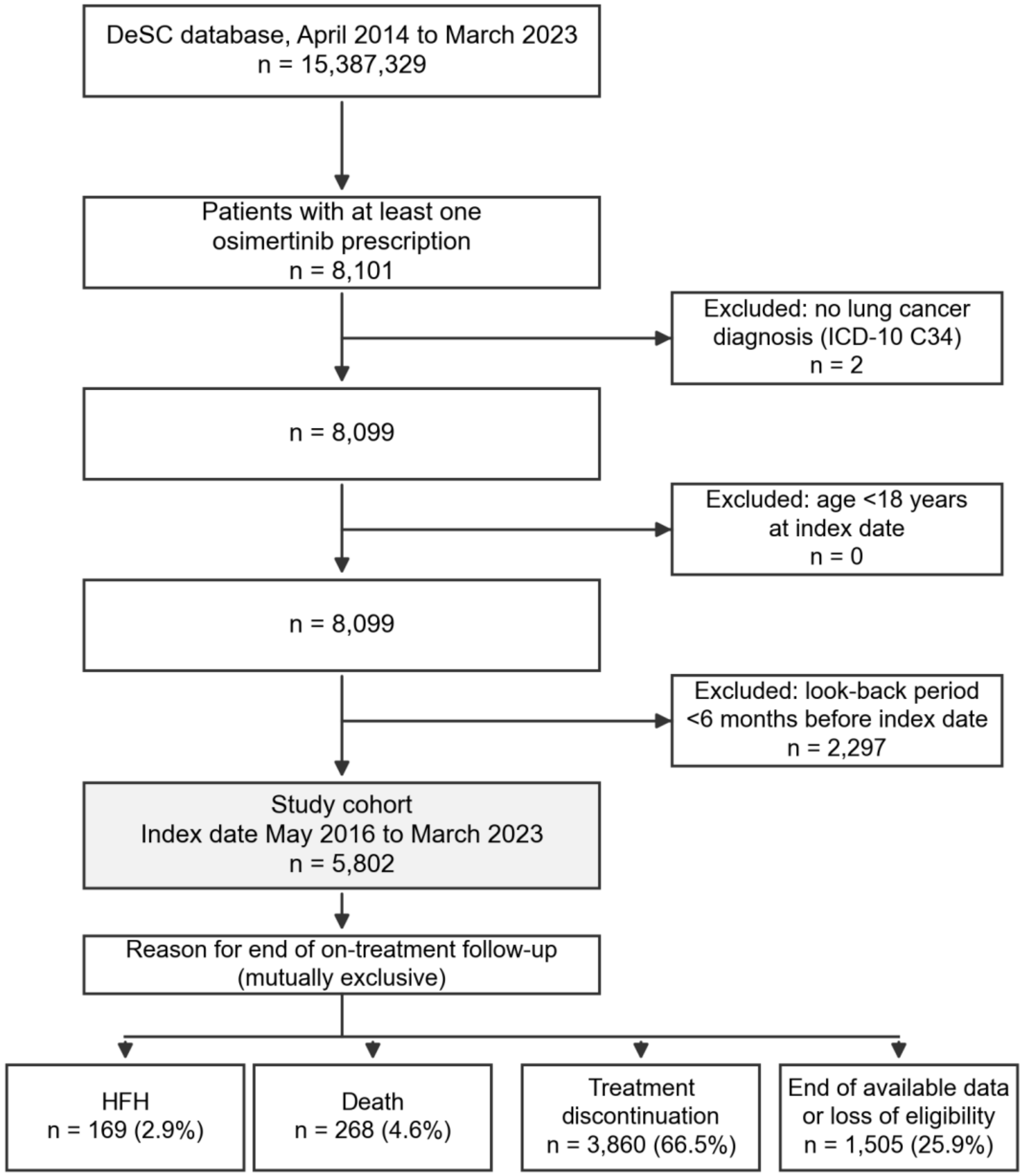

The four categories include 122 patients flagged as deceased with an uninterpretable eligibility end date, who were not counted as deaths: treatment discontinuation 111, HFH 8, end of available data 3.

Figure S2. Flow of participants. Exclusions are shown in the order in which they were applied. The lower boxes give the reason for the end of on-treatment follow-up; end of available data includes loss of insurance eligibility for reasons other than death. HFH, heart failure hospitalization.
